# User Experience and Extended Technology Acceptance Model in Commercial Health Care App Usage Among Patients With Cancer: Mixed Methods Study

**DOI:** 10.2196/55176

**Published:** 2024-12-18

**Authors:** Ye-Eun Park, Yae Won Tak, Inhye Kim, Hui Jeong Lee, Jung Bok Lee, Jong Won Lee, Yura Lee

**Affiliations:** 1 Department of Information Medicine Asan Medical Center University of Ulsan College of Medicine Seoul Republic of Korea; 2 Graduate Program of Industrial Pharmaceutical Science Yonsei University Seoul Republic of Korea; 3 MediRama Seoul Republic of Korea; 4 Department of Clinical Epidemiology and Biostatistics Asan Medical Center University of Ulsan College of Medicine Seoul Republic of Korea; 5 Division of Breast Surgery, Department of Surgery Asan Medical Center University of Ulsan College of Medicine Seoul Republic of Korea

**Keywords:** mHealth, user experience, cancer, technology acceptance model, structural equation modeling, health care app, mixed-method study, medical care, digital health care, cancer survivors, disparities, health status, behavioral intervention, clinician

## Abstract

**Background:**

The shift in medical care toward prediction and prevention has led to the emergence of digital health care as a valuable tool for managing health issues. Aiding long-term follow-up care for cancer survivors and contributing to improved survival rates. However, potential barriers to mobile health usage, including age-related disparities and challenges in user retention for commercial health apps, highlight the need to assess the impact of patients’ abilities and health status on the adoption of these interventions.

**Objective:**

This study aims to investigate the app adherence and user experience of commercial health care apps among cancer survivors using an extended technology acceptance model (TAM).

**Methods:**

The study enrolled 264 cancer survivors. We collected survey results from May to August 2022 and app usage records from the app companies. The survey questions were created based on the TAM.

**Results:**

We categorized 264 participants into 3 clusters based on their app usage behavior: short use (n=77), medium use (n=101), and long use (n=86). The mean usage days were 9 (SD 11) days, 58 (SD 20) days, and 84 (SD 176) days, respectively. Analysis revealed significant differences in perceived usefulness (*P*=.01), interface satisfaction (*P*<.01), equity (*P*<.01), and utility (*P*=.01) among the clusters. Structural equation modeling indicated that perceived ease-of-use significantly influenced perceived usefulness (β=0.387, *P*<.01), and both perceived usefulness and attitude significantly affected behavioral intention and actual usage.

**Conclusions:**

This study showed the importance of positive user experience and clinician recommendations in facilitating the effective usage of digital health care tools among cancer survivors and contributing to the evolving landscape of medical care.

## Introduction

As the paradigm of medical care has shifted toward prediction, prevention, personalization, and participation, digital health care has emerged as a promising tool for managing health-related issues [1-3]. Digital health care has the potential to become an effective aid for long-term follow-up care among cancer survivors, contributing to increased survival rates [4-8]. While the evidence for the effectiveness of digital health care has been accumulated through well-designed clinical studies, there are potential barriers. Compared to 78% of cancer survivors who are aged 60 years or older, smartphone ownership and internet usage are lower among older people [9-12]. Also, users’ digital literacy, socioeconomic status, and user interface (UI) of apps are hurdles to successful digital health intervention [13-17].

Though long-term use of health care apps has been linked to better outcomes, many commercial health apps face challenges in user retention [18,19]. To understand how we could make patients to use health care applications effectively, it is important to identify the factors influencing the user’s adoption of digital interventions. The technology acceptance model (TAM) provides a theoretical framework that identifies and measures various factors influencing users’ adoption and usage of digital technology; it is a useful tool for understanding technology acceptance [20,21]. Furthermore, accessibility, convenience, and self-efficacy are key facilitators for adopting digital health applications; thus, it is essential to examine the impact of the ability of patients with cancer to use mobile technologies and their health status on the usage of mobile health interventions [5,22,23].

There have been many studies on the process of developing apps for specific health problems, considering patients’ needs and user-centered design [24]. However, from the perspective of older patients with cancer who use conventional commercial health management apps, this consideration is far from a real-world situation. In addition, when recommending or promoting commercial health apps to improve the quality of life of patients with cancer, there is a lack of evidence on what factors can make patients use them better and find them more helpful [8,25].

Therefore, the objective of this study is to investigate the association between user characteristics, user experience, and the level of compliance with commercial smartphone health apps among patients with cancer. We analyzed the relationship between application usage logs and survey data to devise an extended TAM model for effective elucidation of the relationships among the variables that contribute to the actual usage of the apps.

## Methods

### Study Design

This study aimed to provide insights into the factors that affect digital health care app acceptance and sustained use, proposing an enhanced TAM. The survey included various categories such as socioeconomic demographics (D), usual usage of smartphones, health management (HM) habits, user experience (app usability [Us]), interface satisfaction (IS), equity (Eq), utility (Ut), enjoyment (Ej), active willingness to use the app (AW), attitude toward usefulness (AU), information management (IM), preference for human interaction (HI) via app, willingness to continuous use (CU), preference of app feature (PF), and external motivation given by researchers (ie, attending physician of the study subject). The study enrolled 264 patients, part of a randomized controlled trial (RCT) involving 960 cancer survivors (breast, colorectal, or lung cancer), excluding a control group and accounting for a 20% dropout rate, from a pool of patients who had used the app for at least 6 months, aimed at assessing the impact of mobile Health apps on recovery. The description of the features of the 4 apps used in the prospective RCT is provided in Multimedia Appendix 1. And we employed structural equation modeling (SEM) to test hypotheses regarding the influence of motivation, perceived usefulness, and perceived ease of use on users' attitudes and behavioral intentions toward the app.

### Questionnaire Design

The development of the survey was based on the TAM and prior research exploring perceived ease-of-use and perceived usefulness, commonly used in studies related to mobile health care, smartphone adoption, and continuous usage intention. The survey categories and items were selected based on previous studies investigating the factors influencing the acceptance of digital health care products and services [26-36]. The set of variables and reference articles relating to each TAM variable, category, and operational definition are shown in Table S1 in Multimedia Appendix 1. The final survey categories were as follows: D, use of smartphone, HM, Us, IS, Eq, Ut, Ej, AW, AU, IM, HI, CU, PF, and motivation (M). The matching of each category with TAM variables is shown in Figure S1 in Multimedia Appendix 1.

The response formats for each survey item were measured according to the nature of the questionnaire. These formats included the 5-point Likert scale (1=strongly disagree to 5=strongly agree), yes or no responses, single-choice selections, multiple-choice selections, and open-ended responses. The survey was created as a web-based individual survey link and sent to respondents on their devices (smartphones or PCs). For a complete description of the survey, see Multimedia Appendix 1.

### Enrollment Process

Among the 960 participants involved in a previous protocol study, a control group of 240 individuals who did not use the app was excluded [37-39]. During the recruitment period, patients who presented for medical visits after a minimum of 6 months since the initiation of app usage were approached to obtain research participation consent. The sample size for the research participants was calculated based on the survey sample size formula [40]. With a confidence level of 95% and a margin of error of 5%, considering the population proportions for breast, colorectal, and lung cancer (33%), the appropriate sample size was determined to be 252 out of the total population of 720. Additionally, hypothesis testing was conducted using Cronbach α with a minimum acceptable value of .95, an expected value of .97, a significance level of .05 (2-tailed), a power of 80%, an expected dropout rate of 5%, and a total of 90 survey items. The sample size calculator determined that a sample size of at least 63 respondents was required to achieve valid reliability.

**Figure 1:**
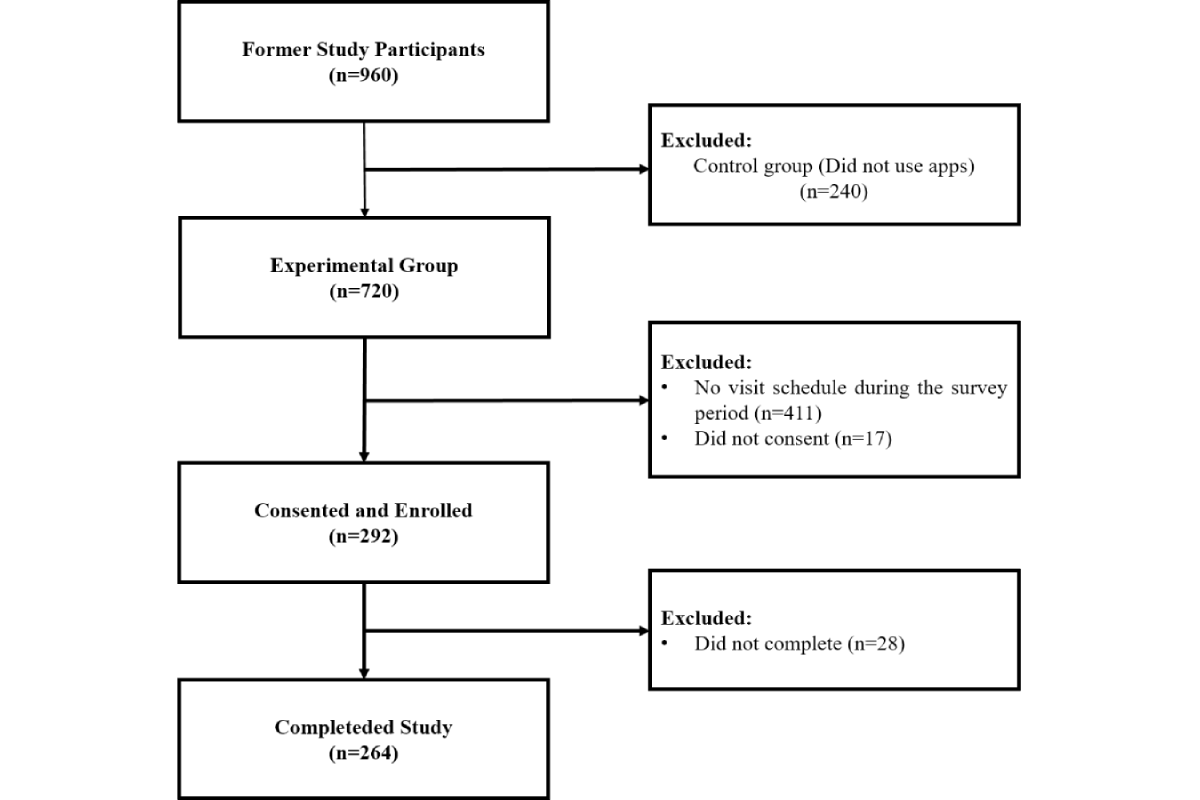
Flow diagram summarizing the enrolment process.

The survey was conducted from May 17, 2022, to August 26, 2022. A dropout rate of 20% was considered during patient recruitment, and a total of 309 patients were available for enrollment during the recruitment period. Among them, 17 (5.5%) patients who refused to participate in the survey were excluded. After receiving the survey link, the participants were requested to complete the survey within the designated research period. Participants who failed to complete the survey within 1 week received a reminder, and those who did not finish it within an additional week were deemed to have dropped out of the study. Out of 292 (94.5%) participants who agreed to participate in the survey, 28 (9.1%) did not complete it during the study period and were excluded. The final number of participants who completed the survey was 264 (85.4%; Figure 1).

### Data Collection

The app used in the study is commercially available and can be downloaded from the Korean App Store. In the case of paid apps, the research funds were used to cover the costs of using the app [37-39]. The app usage data were collected from the app development company, with the consent of the research participants. Information collected included app access timestamps and frequency of access.

### Data Analysis

#### Questionnaire Result Analysis

We used chi-square cross-analysis to calculate the *P* values for survey items with binary responses. For survey items with continuous variable responses, such as Likert scale responses, we used the Wilcoxon rank sum test to calculate the p-values. In both cases, we compared 2 groups at a time rather than analyzing all 3 groups (short use, medium use, and long use) simultaneously. The data analysis was performed using R (version 4.3.0). For all survey items, excluding the responses related to continuous variables and open-ended responses, the responses were coded using numerical values without assigning any inherent meaning to the magnitude of the numbers. These coded responses were then used for calculations as nominal variables.

For binary survey responses (yes or no), we coded the 264 participants’ answers as follows: positive experiences as 1 and negative experiences as 0. We then calculated the ratio of positive responses within each category based on the total number of questions in that category. Participants were grouped as high or low within each category based on their scores compared to the category average. If an individual’s score in a specific category exceeded the category average, they were categorized as high; otherwise, they were classified as low. Participants were labeled as positive or high if more than half of their category groups were high; otherwise, they were classified as negative or low.

#### Actual App Usage Data Analysis

The participants’ actual app usage data were obtained using the dates and times when the app was opened for each usage during a period of 180 days. By counting the number of app access days for each patient, 3 components were calculated for K-means clustering: mean number of use, total term usage, and number of app access days [41]. The mean number of use was calculated as an average of app usage days based on the first usage date of the app. Total term usage, measured in days, was the difference between the dates of the first and last app access. The number of app access days refers to the count of days a patient accessed the app within the 180 days starting from their initial access date.

We classified patients into 3 clusters: short use, medium use, and long use [42]. The short-use group comprised patients who only used the app for 12 weeks during the intervention period. The medium-use group comprised patients who used the app for more than 12 weeks but did not use it persistently beyond that period. The long-use group consisted of patients who used the app continuously. The data analysis was performed using Python (version 3.8.5; Python Software Foundation).

#### Structural Equation Modeling

**Figure 2:**
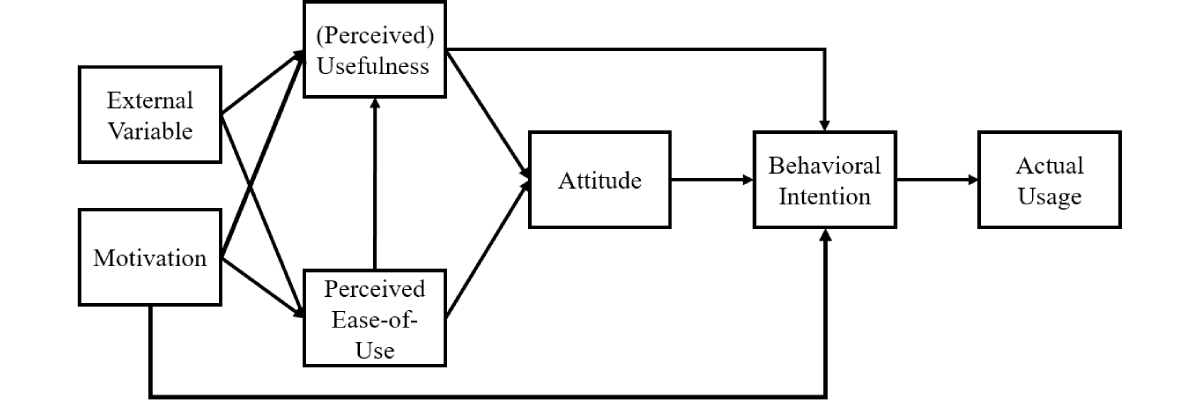
Enhanced TAM (technology acceptance model; tailored for a digital health care app).

Descriptive statistics and SEM using latent variable analysis were performed using the Lavaan software (version 0.6-15; Ghent University) in R (version 4.3.0; R Foundation for Statistical Computing). As this study’s sample was from 264 patients, the sample size was adequate for model analysis [43]. A *P* value less than .05 was considered statistically significant. In our proposed model, we introduced a new node called “Motivation” to the existing TAM [44] and hypothesized that it could influence (perceived) usefulness, perceived ease-of-use, and behavioral intention. Moreover, perceived usefulness was changed to (perceived) usefulness, as the apps were commercially available and had already demonstrated their Ut. The “Motivation” node represented how the participants felt while participating in the clinical study using the app. The schematic representation of the proposed theoretical model is presented in Figure 2. Moreover, the comparative fit index, Tucker-Lewis index, root mean square error of approximation, and standardized root mean square residual were used to identify the fit of the theoretical model.

Therefore, through the SEM, we attempted to answer various hypotheses as follows: motivation will positively affect perceived usefulness and perceived ease of use. Moreover, external variables will positively affect perceived usefulness and perceived ease of use. The perceived ease-of-use of application users will positively affect their (perceived) usefulness, their attitude toward the application, and their behavioral intention to continue using the application. The (perceived) usefulness of application users will positively affect their attitude toward the application and their behavioral intention to continue using the application. The attitude of application users toward the application will positively affect their behavioral intention to continue using the application. Finally, the behavioral intention of application users will positively affect their actual usage of the application.

### Ethical Considerations

This study was approved by the institutional review board of Asan Medical Center, Korea (IRB 2021-1631) and adhered to relevant ethical guidelines. Informed consent was obtained from all participants before their involvement in the study, with assurances of anonymity and confidentiality provided. Participants were briefed on the study's objectives and how the collected data would be used. Additionally, stringent measures were in place to protect the privacy and confidentiality of study data, including secure storage within the hospital premises. Each participant received a US $7 gift voucher as compensation for their time.

## Results

### Categorizing Patients According to Their App Usage Behavior Using K-Means Clustering

**Figure 3:**
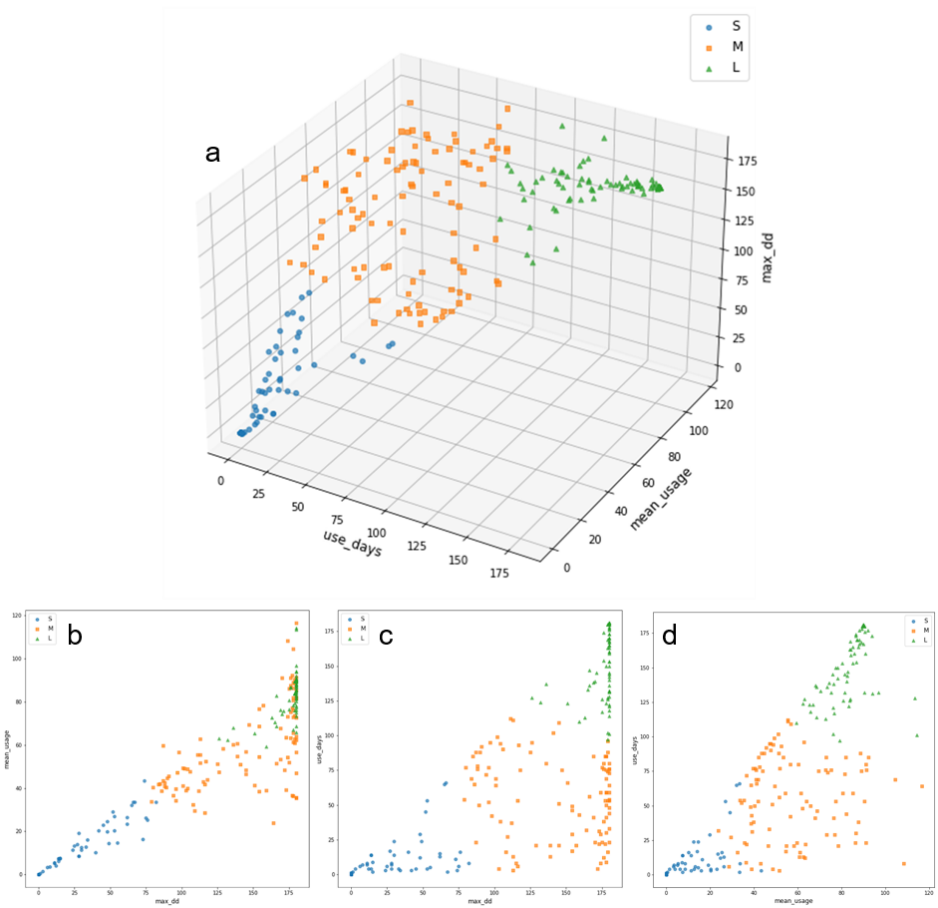
Results of K-means clustering on sample (n=264). Results of K-means clustering for (a) 3 components: number of app-access days (use_days), mean number of use (mean_usage), and total term of use (max_dd); (b) max_dd–mean_usage perspective; (c) max_dd–use_days perspective; and (d) mean_usage–use_days perspective.

We classified the participants into 3 clusters based on their app usage behavior. The clusters were labeled S, M, or L (short use, medium use, and long use, respectively) as shown in Figure 3. There were 77 short-use patients: the average of their mean number of use was 9 (SD 11) days, the mean of total term of use was 21 (SD 21) days, and the mean number of app access days was 7 (SD 14) days. The means for the 101 medium-use patients were 58 (SD 20) days for mean number of use, 144 (SD 36) days for total term of use, and 55 days for number of app access days. Finally, the 86 long-use patients had the following means: 84 days for mean number of use, 176 days for total term of use, and 154 days for number of app access days.

Gender distribution significantly varied across groups (*P*=.02), with a higher proportion of males in the short group (40.3%) compared to the long group (19.8%). The median age ranged from 54 to 57 years across groups (*P*=.08). Participants from urban areas were more prevalent in the long group (60.5%), while those from rural areas were less represented (*P*=.06). Education levels differed significantly across the group (*P*=.04), where a higher proportion of individuals in the short group had a more middle school graduate or lower compared to the medium and long groups. Even though high school graduates were fairly evenly distributed across the groups, university graduates or higher were most prevalent in the medium group. Marital status did not differ significantly across groups *P*=.27). App usage varied notably (*P*<.01), with higher usage of Noom in the long group (52.3%), while Walkon and Second Doctor were more common in the medium group (46.5% and 28.7%, respectively). Breast cancer was the most common cancer type, especially in the long group (51.2%), with significant differences in cancer type distribution across groups (*P*=.03). Clinical stage distribution showed no significant differences (*P*=.39), though stage 1 was most prevalent in all groups, particularly in the long group (66.3%). The details of the demographics for each group are available at Table 1.

**Table 1:** Demographics of the participants in respect to their term use.

Variables		Total (N=264)	Short (n=77)	Medium (n=101)	Long (n=86)	*P* value	
**Gender, n (%)**							*.02^a^*
	Male	78 (29.5)	31 (40.3)	30 (29.7)	17 (19.8)		
	Female	186 (70.5)	46 (59.7)	71 (70.3)	69 (80.2)		
Age (years), median (range)		54.0 (21-79)	57.0 (30-79)	54.0 (21-74)	54.5 (21-77)	.08	
**Residence, n (%)**							.06
	Urban	132 (50.0)	35 (45.5)	45 (44.6)	52 (60.5)		
	Rural	132 (50.0)	42 (54.5)	56 (55.4)	34 (39.5)		
**Education, n (%)**							*.04*
	Middle school graduate or lower	21 (7.9)	11 (14.3)	5 (5.0)	5 (5.9)		
	High school graduate	87 (33.0)	27 (35.1)	28 (27.7)	32 (37.2)		
	University graduate or higher	156 (59.1)	39 (50.6)	68 (67.3)	49 (56.9)		
**Marital status, n (%)**							.27
	Married (having a spouse)	221 (83.7)	3 (3.9)	4 (4.0)	10 (11.6)		
	Never married	17 (6.4)	64 (83.1)	90 (89.1)	67 (77.9)		
	Other	26 (9.8)	10 (13.0)	7 (6.9)	9 (10.5)		
**Monthly household income (US $), n (%)**							.74
	Less than 1150-2300	15 (5.7)	5 (6.5)	5 (5.0)	5 (5.8)		
	2300-4600	106 (40.2)	31 (40.3)	44 (43.6)	31 (36.0)		
	4600 and above	132 (50.0)	36 (46.8)	49 (48.5)	47 (54.7)		
	Does not wish to answer	11 (4.2)	5 (6.5)	3 (3.0)	3 (3.5)		
**Used app, n (%)**							*<.01*
	Walkon	97 (36.7)	17 (22.1)	47 (46.5)	33 (38.4)		
	Noom	81 (30.7)	12 (15.6)	24 (23.8)	45 (52.3)		
	Second Doctor	59 (22.3)	22 (28.6)	29 (28.7)	8 (9.3)		
	Efilcare	27 (10.2)	26 (33.8)	1 (1.0)	0 (0)		
**Cancer type, n (%)**							*.03*
	Breast cancer	114 (43.2)	22 (28.6)	48 (47.5)	44 (51.2)		
	Colon cancer	64 (24.2)	25 (32.5)	26 (25.7)	13 (15.1)		
	Lung cancer	86 (32.6)	30 (39.0)	27 (26.7)	29 (33.7)		
**Clinical stage (p-stage), n (%)**							.39
	Stage 1	154 (58.3)	43 (55.8)	54 (53.5)	57 (66.3)		
	Stage 2	62 (23.5)	21 (27.3)	24 (23.8)	17 (19.8)		
	Stage 3	31 (11.7)	10 (13.0)	13 (12.9)	8 (9.3)		
	Stage 4	17 (6.4)	3 (3.9)	10 (9.9)	4 (4.7)		

^a^The italicized values represent statistically significant (*P*<.05) comparisons.

### Relationship Between App Usage and TAM Factors

Next, we conducted a comparative analysis using the survey categories corresponding with the TAM structure variables. We calculated the average positive response scores for each category and classified individuals into high or low groups based on whether their scores were above or below the mean score, respectively. Furthermore, we compared the proportions of the short-use, medium-use, and long-use cluster groups between the High and Low groups (Table 2). Moreover, the overall survey response groups are available in Table S2 in Multimedia Appendix 1.

**Table 2:** Distribution of clustering groups by survey category according to the technology acceptance model (TAM) structure.

Technology acceptance model (TAM) structure (elements, questionnaire, and response result groups)				Total (N=264)		Usage cluster category							*P* value	
						Short (n=77)		Medium (n=101)		Long (n=86)				
**Perceived usefulness, n (%)**														
	**Utility**													*.01^a^*
		High	196 (74.2)		48 (62.3)		78 (77.2)		70 (81.4)					
		Low	68 (25.8)		29 (37.7)		23 (22.8)		16 (18.6)					
	**Enjoyment**													.54
		High	120 (45.5)		23 (29.9)		45 (44.6)		52 (60.5)					
		Low	144 (54.5)		54 (70.1)		56 (55.4)		34 (39.5)					
**Perceived ease-of-use, n (%)**														
	**Interface satisfaction**													*<.01*
		High	197 (74.6)		45 (58.4)		78 (77.2)		73 (84.9)					
		Low	67 (25.4)		32 (41.6)		23 (22.8)		13 (15.1)					
	**Equity**													*<.01*
		High	193 (73.1)		44 (57.1)		77 (76.2)		72 (83.7)					
		Low	71 (26.9)		33 (42.9)		24 (23.8)		14 (16.3)					
	**Usability**													*.01*
		High	183 (69.3)		40 (51.9)		75 (74.3)		68 (79.1)					
		Low	81 (30.7)		37 (48.1)		26 (25.7)		18 (20.9)					
**Attitude, n (%)**														
	**Active willingness to use the app**													.92
		High	115 (43.6)		17 (22.1)		46 (45.5)		52 (60.5)					
		Low	149 (56.4)		60 (77.9)		55 (54.5)		34 (39.5)					
	**Attitude toward usefulness**													.10
		High	189 (71.6)		45 (58.4)		75 (74.3)		69 (80.2)					
		Low	75 (28.4)		32 (41.6)		26 (25.7)		17 (19.8)					
	**Information management**													.32
		High	168 (63.6)		41 (53.2)		65 (64.4)		62 (72.1)					
		Low	96 (36.4)		36 (46.8)		36 (35.6)		24 (27.9)					
	**Human interaction**													*.03*
		High	135 (51.1)		34 (44.2)		47 (46.5)		54 (62.8)					
		Low	129 (48.9)		43 (55.8)		54 (53.5)		32 (37.2)					

^a^The italicized values represent statistically significant (*P*<.05) comparisons.

The short-use clustering group exhibited higher proportions of low respondents than the other groups in all categories. Furthermore, among the categories corresponding to perceived usefulness and perceived ease-of-use, except for Ej, significant differences were observed among the cluster groups in all categories (Us: *P*=.01; IS: *P*<.01; E: *P*<.01; Ut: *P*=.01). The proportion of low respondents among the overall survey participants was 55% in the Ej category. The *P* values for the individual group comparisons between the high or low groups and short-use or medium-use- or long-use cluster groups have been provided in Table S3 in Multimedia Appendix 1.

### Structural Equation Modeling

**Figure 4:**
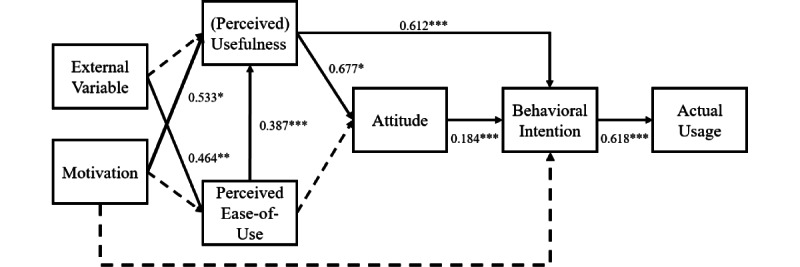
Results from structural equation modeling show the relevance among TAM (technology acceptance model) variables. Standardized estimates of the relationships between variables are indicated by solid lines. Significant relationships are denoted by *(*P*<.05), **(*P*<.01), and ***(*P*<.001), while broken lines represent non-significant relationships. The solid lines represent observed significant relationships, confirming the hypothesized connections.

For this extended TAM, the fit of data was favorable; comparative fit index: 0.905, Tucker-Lewis index: 0.890, root mean square error of approximation: 0.052, and standardized root mean square residual: 0.071. The final structural model with estimated standardized coefficients is presented in Figure 4, and the estimation results of the hypotheses are shown in Table S4 in Multimedia Appendix 1.

The path from external variables to (perceived) usefulness was not statistically significant (β=–0.061, *P*=.272). However, there was a significant and positive path from external variables to perceived ease-of-use (β=0.464, *P*=.004). While there was a significant path from motivation to (perceived) usefulness (β=0.533, *P*=.003), the paths from motivation to perceived ease-of-use (β=0.293, *P*=.401) and behavioral intention (β=–0.197, *P*=.201) were not statistically significant. Perceived ease-of-use significantly influenced (perceived) usefulness (β=0.387, *P*<.01). Perceived usefulness, in turn, had a significant positive effect on attitude (β=.677, *P*=.02). Furthermore, (perceived) usefulness had a significant positive effect on behavioral intention (β=0.612, *P*<.01). Attitude significantly influenced behavioral intention (β=0.184, *P*<.01). Finally, behavioral intention had a significant positive effect on actual usage (β=0.618, *P*<.01).

## Discussion

### Principal Findings

We analyzed questionnaire responses and app usage log data of patients with cancer who used a commercial smartphone health care app as an intervention in a prospective RCT, stratified users by whether they used the app faithfully or not, and identified factors affecting app usage. When we grouped patients using app usage logs, we considered not only total usage duration but also amount and access frequency, and the group (S) who used the app the least actively showed a maximum usage duration close to 12 weeks, the original RCT intervention period. Because the researchers did not discourage or encourage use after the 12-week intervention period, the above results suggest that the study setting had a meaningful effect on the continuation of app usage among the study subjects. Though we classified patients into 3 groups, “short use, medium use, and long use,” this is meaningful because the groups do not simply refer to the length of the maximum usage period but also to whether they used the app actively and loyally (Table S2 in Multimedia Appendix 1).

The analysis of the study population revealed notable differences among participants grouped by the duration of health intervention app usage. Gender distribution was significantly different across groups, where females may be more inclined to engage in prolonged use of health-related apps, potentially reflecting greater health-seeking behavior or adherence to digital interventions among women [45]. Residence patterns showed that participants from big cities were more likely to be in the long group, possibly due to better access to digital resources or a higher level of digital literacy in urban areas. App usage patterns varied considerably, with Noom being most popular in the long-duration group, while Walkon and Second Doctor were more common among those with medium-duration usage. This variation in app preference may be related to the specific features, usability, or perceived effectiveness of the apps, influencing users' commitment over time. Cancer type distribution also differed significantly, with patients with breast cancer more likely to be in the long-duration group [46].

In the category with TAM variables, significant differences among the cluster groups were observed in categories related to perceived usefulness and perceived ease of use, except for Ej. SEM results revealed that external variables significantly influenced perceived ease of use but not perceived usefulness. Motivation significantly influenced perceived usefulness but did not significantly affect perceived ease of use or behavioral intention. Perceived ease of use significantly influenced perceived usefulness, which in turn significantly affected attitude and behavioral intention. Attitude significantly influenced behavioral intention, which then had a significant positive effect on actual usage.

### Interpretation Within the Context of Wider Literature

Despite continuing skepticism about commercial smartphone applications' effectiveness for HM, there is no doubt that smartphone apps have the potential to drive improved health through behavioral change by always being beside users [47]. Through this mixed methods study, we determined that the factors influencing the consistent use of apps are not solely dependent on user characteristics, such as age, gender, and typical smartphone usage experience [48]. A physician's recommendation, validated by an assessment tool and model, can exert a notable influence as external motivation. Also, daily smartphone use, although unmodifiable, aids in detecting non-compliance within high-risk groups using digital health tools [49]. Perceptions of ease-of-use and usefulness significantly influence app usage, highlighting the crucial role of UI design and app content in digital HM. The study emphasizes the importance of doctors understanding digital health apps for making effective recommendations. Proficiency in smartphone use correlates with high perceived ease-of-use, consistent with structural equation results. Further research is needed to determine if familiarity with smartphone interaction increases positive responses to notifications or messages.

Participant demographics such as age, gender, residence, education, and income did not significantly correlate with app usage experience. However, being female, below the median age, and having an income exceeding US $4600 were key characteristics of participants inclined to use apps, aligning with studies on app retention [50]. While smartphone ownership has reduced digital inequality, our findings suggest that factors associated with complex UI vulnerability still influence app usage.

Distinct differences were observed among the 3 groups regarding usage duration across all 3 criteria. Analyzing TAM factors in these groups revealed notable differences in IS and Eq. Us, Ut, AU, and HI also varied among the groups. Notably, IS and Eq exhibited the most significant differences, with higher percentages linked to increased app usage. This indicates that low Us stemmed from the challenging user experience while using the app. Previous studies have also indicated that a user-friendly interface offering simplicity and automation encourages the use of health care apps, particularly among older patients [51-54]. Our findings align with these studies, as patients who reported enjoying the app’s interactions and user-friendly interface were more prevalent in the group with higher app usage.

Overall, the significant connections positively affected the other variables. Except for the connections from external variables to (perceived) usefulness, motivation to perceived ease-of-use, and motivation to behavioral intention, the remaining connections from the improved TAM exhibited the following significant and positive paths: external variables to perceived ease-of-use, motivation to (perceived) usefulness, perceived ease-of-use to (perceived) usefulness, (perceived) usefulness to attitude, attitude to behavioral intention, and behavioral intention to actual usage. The presented findings align with existing research on mobile services and portable electronic devices. Kuo and Yen [55] investigated 3rd generation mobile value-added services and found a positive and significant relationship between perceived usefulness, behavioral intention, and attitude. Similarly, Chen and Chen [56] demonstrated a significant link between perceived ease-of-use and attitude among travelers using global positioning system devices.

We can better understand how different aspects correlate with the app's actual usage by closely examining the extended TAM at a more granular level. In terms of demographic data, the extended TAM revealed that female patients exhibited higher levels of app engagement compared to males. Furthermore, younger patients displayed more frequent and proficient app usage. This trend aligns with previous research findings, which have consistently highlighted the greater comfort and familiarity that female and younger individuals have with mobile apps, leading to prolonged usage [57,58]. This might be attributed to the fact that women tend to use more health apps related to fertility and pregnancy, indicating their openness to other health care apps as well [58]. Additionally, patients with higher education and income levels exhibited elevated app usage rates. Furthermore, individuals who reported more extensive smartphone usage in their current or previous professional roles were more inclined to use the app extensively. This association can be attributed to the increased comfort and familiarity that smartphone-familiar individuals have with mobile technology. Such users are more predisposed to engage with the app, as they already integrate smartphones more prominently into their daily routines than those without a history of smartphone use in their professional roles.

Digital interventions for clinical research are primarily designed for research purposes, with acknowledged limitations when using commercialized smartphone apps [59]. While some digital therapeutics and HM apps demonstrate effects proven through clinical studies, there is a lack of consistent indicators for app use compliance, even in research apps. Results may differ between clinical studies and real-world scenarios, as participants in clinical studies might not actively choose their application. Recognizing patients more as consumers, clinician recommendations play a crucial role in app retention, boosting confidence and adherence [60]. This underscores the potential of clinician recommendations to enhance adherence to digital health solutions.

Compared to other chronic diseases, the relationship between the patient with cancer and oncologist (surgical oncologist) is especially important in the dynamic treatment process and decision-making [61,62]. Previous research has attempted to define the patient with cancer–physician relationship in various ways [63]. Palmer Kelly et al [64] explained the patient with cancer–physician relationship with attachment model, and an appropriate attachment relationship between patients with cancer and physicians is associated with a better quality of life. Our findings support the idea that the patient-physician relationship can influence app acceptance and serve as a potential factor for increasing compliance with digital interventions. Physical activity is encouraged to cope with anxiety and depression in patients with cancer, and outdoor physical activity in patients with cancer can be promoted by organized activities [65,66]. Therefore, we can expect that if the use of digital interventions is encouraged by his or her oncologist (surgical oncologist), the effect on the quality of life of patients could be further improved.

### Strengths and Limitations

To our knowledge, this is the first study that integrated user experience evaluation in commercial health care apps for cancer survivors, examining their adherence through usage log data analysis. Moreover, this is the first study to use SEM to extend the TAM to be well suited in a mobile health context. The integration of these 2 approaches enables a comprehensive understanding of the factors influencing the CU intention of digital health care applications among patients with cancer, including the effects of health status and demographics. Our study provides insights into the key determinants of mobile health care app usage among patients with cancer, emphasizing the role of user experience, clinician recommendations, and the potential to bridge digital health care inequalities.

The traditional TAM faces limitations in capturing recent technological environment changes and fails to reflect the diverse information acquisition paths for consumers [67,68]. The classical TAM is criticized for not accounting for users' voluntary choice of technology, particularly in the context of HM apps where users may not opt for the technology voluntarily [69]. In addition, since this study targeted only those who could use a smartphone, it was difficult to determine how noncompliance with digital intervention is related to digital inequality [70]. All the participants in this study were from Korea, which could limit its generalizability. However, despite the homogeneity of race and culture in Korea compared to countries such as the United States, the characteristics of the patients would not pose a significant generalizability problem. The age of breast cancer onset varies by ethnicity, with Asian women having a younger age of breast cancer onset. In addition, breast cancer has a different peak onset age than colon and lung cancer, which may bias the results of this study [71,72]. However, this difference is not substantial enough to affect the study's overall applicability.

### Conclusions

This study highlights the critical factors influencing cancer survivors' adherence to commercial health care apps, emphasizing the pivotal role of external motivation, particularly physician recommendations. Key determinants include perceived ease-of-use and usefulness, highlighting the significance of effective UI design. SEM reveals positive paths from motivation to perceived usefulness and from perceived usefulness to attitude and behavioral intention, impacting actual app usage. These findings stress the importance of positive user experience and clinician recommendations in facilitating the effective usage of digital health care tools among cancer survivors and contributing to the evolving landscape of medical care.

## Appendix

Multimedia Appendix 1 Additional tables and figures.

## Abbreviations

AU: attitude toward usefulness
AW: active willingness to use the app
CU: continuous use
D: demographics
Ej: enjoyment
Eq: equity
HI: human interaction
HM: health management
IM: information management
IS: interface satisfaction
M: motivation
PF: preference of app feature
RCT: randomized controlled trial
SEM: structural equation modeling
TAM: technology acceptance model
UI: user interface
Us: usability
Ut: utility

## Data Availability

The anonymously processed dataset used in this study can be shared by the corresponding author upon reasonable request. Any interested party should submit an appropriate proposal, analysis plan, and protocols for review and approval by the authors. Subsequently, the materials will be submitted for approval to the institutional review board of Asan Medical Center (Seoul, Republic of Korea). Once approved, the data will be made available.

## References

[1] Marcolino MS, Oliveira JAQ, D'Agostino M, Ribeiro AL, Alkmim MBM, Novillo-Ortiz D (2018). The impact of mHealth interventions: systematic review of systematic reviews. JMIR Mhealth Uhealth.

[2] (2018). Smart Healthcare Medical Device Technology/Standard Strategy Report.

[3] Slim K, Selvy M, Veziant J (2021). Conceptual innovation: 4P medicine and 4P surgery. J Visc Surg.

[4] Clauser SB, Wagner EH, Aiello Bowles EJ, Tuzzio L, Greene SM (2011). Improving modern cancer care through information technology. Am J Prev Med.

[5] Hopstaken JS, Verweij L, van Laarhoven CJHM, Blijlevens NMA, Stommel MWJ, Hermens RPMG (2021). Effect of digital care platforms on quality of care for oncological patients and barriers and facilitators for their implementation: systematic review. J Med Internet Res.

[6] Miller KD, Nogueira L, Devasia T, Mariotto AB, Yabroff KR, Jemal A, Kramer J, Siegel RL (2022). Cancer treatment and survivorship statistics, 2022. CA Cancer J Clin.

[7] Penedo FJ, Oswald LB, Kronenfeld JP, Garcia SF, Cella D, Yanez B (2020). The increasing value of eHealth in the delivery of patient-centred cancer care. Lancet Oncol.

[8] Roberts AL, Fisher A, Smith L, Heinrich M, Potts HWW (2017). Digital health behaviour change interventions targeting physical activity and diet in cancer survivors: a systematic review and meta-analysis. J Cancer Surviv.

[9] (2021). Age and Cancer Risk.

[10] Tonorezos E, Devasia T, Mariotto AB, Mollica MA, Gallicchio L, Green P, Doose M, Brick R, Streck B, Reed C, de Moor JS (2024). Prevalence of cancer survivors in the United States. J Natl Cancer Inst.

[11] Laricchia F (2024). Share of adults in the United States who owned a smartphone from 2015 to 2023, by age group. Statista.

[12] Petrosyan A (2024). Distribution of internet users worldwide as of February 2024, by age group. Statista.

[13] Boels AM, Rutten G, Zuithoff N, de Wit A, Vos R (2018). Effectiveness of diabetes self-management education via a smartphone application in insulin treated type 2 diabetes patients - design of a randomised controlled trial ('TRIGGER study'). BMC Endocr Disord.

[14] Madrigal L, Escoffery C (2019). Electronic health behaviors among US adults with chronic disease: cross-sectional survey. J Med Internet Res.

[15] Onyeaka HK, Zambrano J, Longley RM, Celano CM, Naslund JA, Amonoo HL (2021). Use of digital health tools for health promotion in cancer survivors. Psychooncology.

[16] Wilson J, Heinsch M, Betts D, Booth D, Kay-Lambkin F (2021). Barriers and facilitators to the use of e-health by older adults: a scoping review. BMC Public Health.

[17] (2016). Global Diffusion of eHealth: Making Universal Health Coverage Achievable: Report of the third Global Survey on eHealth.

[18] Clement I, Lorenz A, Ulm B, Plidschun A, Huber S (2018). Implementing systematically collected user feedback to increase user retention in a mobile app for self-management of low back pain: retrospective cohort study. JMIR Mhealth Uhealth.

[19] Coughlin JW, Martin LM, Zhao D, Goheer A, Woolf TB, Holzhauer K, Lehmann HP, Lent MR, McTigue KM, Clark JM, Bennett WL (2022). Electronic health record-based recruitment and retention and mobile health app usage: multisite cohort study. J Med Internet Res.

[20] Davis FD (1987). User Acceptance of Information Systems: The Technology Acceptance Model (TAM).

[21] Davis FD (1989). Perceived usefulness, perceived ease of use, and user acceptance of information technology. MIS Q.

[22] Fernandes LG, Devan H, Fioratti I, Kamper SJ, Williams CM, Saragiotto BT (2022). At my own pace, space, and place: a systematic review of qualitative studies of enablers and barriers to telehealth interventions for people with chronic pain. Pain.

[23] Shabir H, D'Costa M, Mohiaddin Z, Moti Z, Rashid H, Sadowska D, Alam B, Cox B (2022). The barriers and facilitators to the use of lifestyle apps: a systematic review of qualitative studies. Eur J Investig Health Psychol Educ.

[24] Eliasen A, Abildtoft MK, Krogh NS, Rechnitzer C, Brok JS, Mathiasen R, Schmiegelow K, Dalhoff KP (2020). Smartphone app to self-monitor nausea during pediatric chemotherapy treatment: user-centered design process. JMIR Mhealth Uhealth.

[25] Srivastava R (2023). Role of smartphone devices in precision oncology. J Cancer Res Clin Oncol.

[26] Cimperman M, Makovec Brenčič M, Trkman P (2016). Analyzing older users' home telehealth services acceptance behavior-applying an extended UTAUT model. Int J Med Inform.

[27] Jang HJ, Noh GY (2017). The effect of health consciousness and playfulness on intention to use tangible fitness game: extended TAM. J Korea Contents Assoc.

[28] Park D, Choi J, Kim D (2015). The influence of health apps efficacy, satisfaction and continued use intention on wearable device adoption: a convergence perspective. J Digit Converg.

[29] Ki YS, Ahn SM, Cho MG, Choi B (2019). An analysis on affecting factors of healthcare applications continuous usage intention and their relationships. J Soc e-Bus Stud.

[30] Kim SS, Ryu S (2011). Structural relationships among factors to adoption of telehealth service. Asia Pac J Inf Syst.

[31] Kim YW, Han S, Kim KS (2018). Determinants of intention to use digital healthcare service of middle and older users. Inf Soc Media.

[32] Lee M, Lee H, Kim Y, Kim J, Cho M, Jang J, Jang H (2018). Mobile app-based health promotion programs: a systematic review of the literature. Int J Environ Res Public Health.

[33] Gao Y, Li H, Luo Y (2015). An empirical study of wearable technology acceptance in healthcare. Ind Manage Data Syst.

[34] Baek M, Choi H, Lee H (2015). Age-specific acceptance intention over wearable smart healthcare device. Korean Acad Assoc Bus Admin.

[35] Lee OH, Ham SW (2017). A study on influence factors of mobile healthcare service using structural equation modeling. J Korea Acad Ind Cooperation Soc.

[36] Wilson EV, Lankton NK (2004). Modeling patients' acceptance of provider-delivered e-health. J Am Med Inform Assoc.

[37] Baek SY, Lee SB, Lee Y, Chung S, Choi CM, Lee HJ, Jo MW, Yun SC, Lee JW (2022). Effects of mobile healthcare applications on the lifestyle of patients with breast cancer: a protocol for a randomized clinical trial. J Breast Cancer.

[38] Kim YI, Park IJ, Kim CW, Yoon YS, Lim SB, Yu CS, Kim JC, Lee Y, Kim H, Chung S, Choi CM, Lee HJ, Kim KW, Ko Y, Yun SC, Jo MW, Lee JW (2022). Lifestyle interventions after colorectal cancer surgery using a mobile digital device: a study protocol for a randomized controlled trial. Medicine (Baltimore).

[39] Lee JH, Jeong JH, Ji W, Lee HJ, Lee Y, Jo MW, Chung S, Yun SC, Choi CM, Lee GD, Lee SW, Lee JW (2022). Comparative effectiveness of smartphone healthcare applications for improving quality of life in lung cancer patients: study protocol. BMC Pulm Med.

[40] SurveyMonkey (2023). Calculating the Number of Respondents You Need. SurveyMonkey.

[41] Kim J, Lim S, Min YH, Shin Y, Lee B, Sohn G, Jung KH, Lee JH, Son BH, Ahn SH, Shin SY, Lee JW (2016). Depression screening using daily mental-health ratings from a smartphone application for breast cancer patients. J Med Internet Res.

[42] Steinley D (2006). K-means clustering: a half-century synthesis. Br J Math Stat Psychol.

[43] Little TD (2013). Longitudinal Structural Equation Modeling.

[44] Davis FD, Bagozzi RP, Warshaw PR (1989). User acceptance of computer technology: a comparison of two theoretical models. Manage Sci.

[45] Fidan E, Çelik S (2021). Factors affecting medical healthcare-seeking behaviours of female patients according to their stage of being diagnosed with breast cancer. Eur J Cancer Care (Engl).

[46] Kong LX, Zhao YH, Feng ZL, Liu TT (2024). Personalized and continuous care intervention affects rehabilitation, living quality, and negative emotions of patients with breast cancer. World J Psychiatry.

[47] Apolinário-Hagen J, Menzel M, Hennemann S, Salewski C (2018). Acceptance of mobile health apps for disease management among people with multiple sclerosis: web-based survey study. JMIR Form Res.

[48] Nunes A, Limpo T, Castro SL (2019). Acceptance of mobile health applications: examining key determinants and moderators. Front Psychol.

[49] Rosenlund M, Kinnunen UM, Saranto K (2023). The use of digital health services among patients and citizens living at home: scoping review. J Med Internet Res.

[50] Rising CJ, Jensen RE, Moser RP, Oh A (2020). Characterizing the US population by patterns of mobile health use for health and behavioral tracking: analysis of the national cancer institute's health information national trends survey data. J Med Internet Res.

[51] van den Berg N, Schumann M, Kraft K, Hoffmann W (2012). Telemedicine and telecare for older patients--a systematic review. Maturitas.

[52] Arnhold M, Quade M, Kirch W (2014). Mobile applications for diabetics: a systematic review and expert-based usability evaluation considering the special requirements of diabetes patients age 50 years or older. J Med Internet Res.

[53] Liew MS, Zhang J, See J, Ong YL (2019). Usability challenges for health and wellness mobile apps: mixed-methods study among mHealth experts and consumers. JMIR Mhealth Uhealth.

[54] Foster M, Xiong W, Quintiliani L, Hartmann CW, Gaehde S (2022). Preferences of older adult veterans with heart failure for engaging with mobile health technology to support self-care: qualitative interview study among patients with heart failure and content analysis. JMIR Form Res.

[55] Kuo YF, Yen SN (2009). Towards an understanding of the behavioral intention to use 3G mobile value-added services. Comput Hum Behav.

[56] Chen CF, Chen PC (2011). Applying the TAM to travelers’ usage intentions of GPS devices. Expert Syst App.

[57] Chowdhury SZ, Stevens S, Wu C, Woodward C, Andrews T, Ashall-Payne L, Leigh S (2023). An age-old problem or an old-age problem? A UK survey of attitudes, historical use and recommendations by healthcare professionals to use healthcare apps. BMC Geriatr.

[58] Paradis S, Roussel J, Bosson JL, Kern JB (2022). Use of smartphone health apps among patients aged 18 to 69 years in primary care: population-based cross-sectional survey. JMIR Form Res.

[59] Sezgin E (2021). Can we use commercial mobile apps instead of research mobile apps in healthcare research?. Front Public Health.

[60] Kang SH, Baek H, Cho J, Kim S, Hwang H, Lee W, Park JJ, Yoon YE, Yoon CH, Cho YS, Youn TJ, Cho GY, Chae IH, Choi DJ, Yoo S, Suh JW (2021). Management of cardiovascular disease using an mHealth tool: a randomized clinical trial. NPJ Digit Med.

[61] Leighl NB, Butow PN, Tattersall MHN (2004). Treatment decision aids in advanced cancer: when the goal is not cure and the answer is not clear. J Clin Oncol.

[62] Kim Y, Winner M, Page A, Tisnado DM, Martinez KA, Buettner S, Ejaz A, Spolverato G, Morss Dy SE, Pawlik TM (2015). Patient perceptions regarding the likelihood of cure after surgical resection of lung and colorectal cancer. Cancer.

[63] Palmer Kelly E, Agne JL, Hyer M, Meara A, Olsen G, Pawlik TM (2019). A systematic review of the methods utilised to measure the relationship between cancer patients and oncologists: implications for future research and practice. Eur J Cancer Care (Engl).

[64] Palmer Kelly E, Tsilimigras DI, Hyer JM, Pawlik TM (2019). Understanding the use of attachment theory applied to the patient-provider relationship in cancer care: recommendations for future research and clinical practice. Surg Oncol.

[65] Andersen BL, Lacchetti C, Ashing K, Berek JS, Berman BS, Bolte S, Dizon DS, Given B, Nekhlyudov L, Pirl W, Stanton AL, Rowland JH (2023). Management of anxiety and depression in adult survivors of cancer: ASCO guideline update. J Clin Oncol.

[66] Lesser IA, Nienhuis CP, Belanger L (2021). Active by nature: exploring cancer survivors' exercise barriers, facilitators, preferences, and psychosocial benefits of engaging in outdoor physical activity. Support Care Cancer.

[67] Nadal C, Sas C, Doherty G (2020). Technology acceptance in mobile health: scoping review of definitions, models, and measurement. J Med Internet Res.

[68] Rahimi B, Nadri H, Lotfnezhad Afshar H, Timpka T (2018). A systematic review of the technology acceptance model in health informatics. Appl Clin Inform.

[69] Holden RJ, Karsh BT (2010). The technology acceptance model: its past and its future in health care. J Biomed Inform.

[70] Cao L, Chongsuvivatwong V, McNeil EB (2022). The sociodemographic digital divide in mobile health app use among clients at outpatient departments in inner Mongolia, China: cross-sectional survey study. JMIR Hum Factors.

[71] Demicheli R, Retsky MW, Hrushesky WJM, Baum M, Gukas ID, Jatoi I (2007). Racial disparities in breast cancer outcome: insights into host-tumor interactions. Cancer.

[72] Stapleton SM, Oseni TO, Bababekov YJ, Hung YC, Chang DC (2018). Race/ethnicity and age distribution of breast cancer diagnosis in the United States. JAMA Surg.

