# Supplementary material for: User Experience and Extended Technology Acceptance Model in Commercial Health Care App Usage Among Patients With Cancer: Mixed Methods Study
Source: J Med Internet Res. 2024 Dec 18;26:e55176. doi: 10.2196/55176 (PMC11694044; doi:10.2196/55176)
Supplement: Multimedia Appendix 1 [file jmir_v26i1e55176_app1.docx]

**Supplementary Table 1.** The set of variables relating to each Technology Acceptance Model (TAM) variable with category and operational definition.

| **TAM** | **Category** | **Variable** | **Operational definition** | **Related question** | **Related research** |
| --- | --- | --- | --- | --- | --- |
| External variables | Demographics (D)  Use of smartphone (US) Health management (HM) | Health interest | Interest in health and health management | US-1,2,3,4 HM-1,2,3,4,5,6 | 2, 6, 8, 9, 11 |
| Perceived ease-of-use | Usability (Us) Interface satisfaction (IS) Equity (E) | Perceived ease-of-use | Perceived ease of use of health management applications in maintaining and managing health | Us-1,2,4 IS-3 | 2, 4, 5, 6, 10, 11 |
|  |  | Design (interface) | Satisfaction with the functionality (interface) and design of health management applications | Us-3,5 IS-1,2 | 6, 8 |
|  |  | Satisfaction | Level of user satisfaction with health management applications | IS-4,5 | 3, 11 |
|  |  | Information quality | Quality of information provided by health management applications | IS-6 | 5, 6, 10 |
| Perceived usefulness | Utility (Ut) Enjoyment (Ej) | Perceived usefulness | Perceived level of efficiency and effectiveness in health management application usage | Ut-1,2,3,4,5 | 2, 4, 5, 6, 7, 10, 11 |
|  |  | Enjoyment | Level of enjoyment experienced by users while using health management applications | Ej-1,2,4 | 2, 4, 8 |
|  |  | Outcome expectations | Level of expected outcomes anticipated from using health management applications | Ej-3 | 1, 8, 9 |
|  |  | Effort expectations | level of expected effort anticipated from users when using health management applications | Ut-4 | 1, 9 |
|  |  | Adoption intention | Intentions and willingness to adopt and use health management applications | Ut-3,4,5 Ej-1,2,3,4 | 2, 8, 9, 10, 11 |
|  |  | Cost-effectiveness | Level of societal/economic cost-effectiveness of using health management applications | Ut-3 | 10 |
| Attitude | Active willingness to use the app (AW) Attitude toward usefulness (AU) Information management (IM) Human interaction (HI) | Intention to use | Level of willingness of prospective users to adopt health management applications in the future | AW-1,2,3,4,5 AU-5 | 1, 3, 4, 6 |
|  |  | Innovativeness | Level of voluntariness of individuals in seeking to use new medical technologies (such as new health management applications) | AU-1,2,3,4  HI-2,3,4  IM-2,3,4 | 4, 5, 6, 9, 10 |
|  |  | Security | Level of sensitivity towards personal information disclosure | IM-1,5  HI-1 | 1, 5, 10 |
|  |  | Health orientation | Willpower of health management application users in pursuit of health | AW-1,2,3,4,5 | 4 |
|  |  | Self-efficacy | Level of self-efficacy obtained through the use of health management applications | AU-2 | 3, 4, 6 |

Notes: TAM: technology acceptance model.

Related research list:

1. Cimperman *et al.* Analyzing older users’ home telehealth services acceptance behavior—applying an Extended UTAUT model. *International journal of medical informatics* (2016).

2. Jang *et al*. The Effect of Health Consciousness and Playfulness on Intention to Use Tangible Fitness Game: Extended TAM. *The Journal of the Korea Contents Association* (2017).

3. Jin *et al*. The Influence of Health Apps Efficacy, Satisfaction and Continued Use Intention on Wearable Device Adoption: A Convergence Perspective. *Journal of Digital Convergence* (2015).

4. Ki *et al*. An Analysis on Affecting Factors of Healthcare Applications Continuous Usage Intention and their Relationships. *Journal of Society for e-Business Studies* (2019).

5. Kim *et al*. Structural relationships among factors to adoption of telehealth service. *Asia pacific journal of information systems* (2011).

6. Kim *et al*. Determinants of Intention to Use Digital Healthcare Service of Middle and Older Users. *Information Society & Media* (2018).

7. Lee *et al.* Mobile app-based health promotion programs: a systematic review of the literature. *International journal of environmental research and public health* (2018).

8. Lee *et al*. An Empirical Study on Acceptance Intention Towards Healthcare Wearable Device. *The Journal of Information Systems* (2016).

9. Baek *et al*. Age-Specific Acceptance Intention over Wearable Smart Healthcare Device. *Korean Journal of Business Administration* (2015).

10. Lee *et al*. A Study on Influence Factors of Mobile Healthcare Service Using Structural Equation Modeling. *Journal of Korea Academia-Industrial cooperation Society* (2017).

11. Wilson *et al*. Modeling patients' acceptance of provider-delivered e-health. *Journal of the American Medical Informatics Association* (2004).


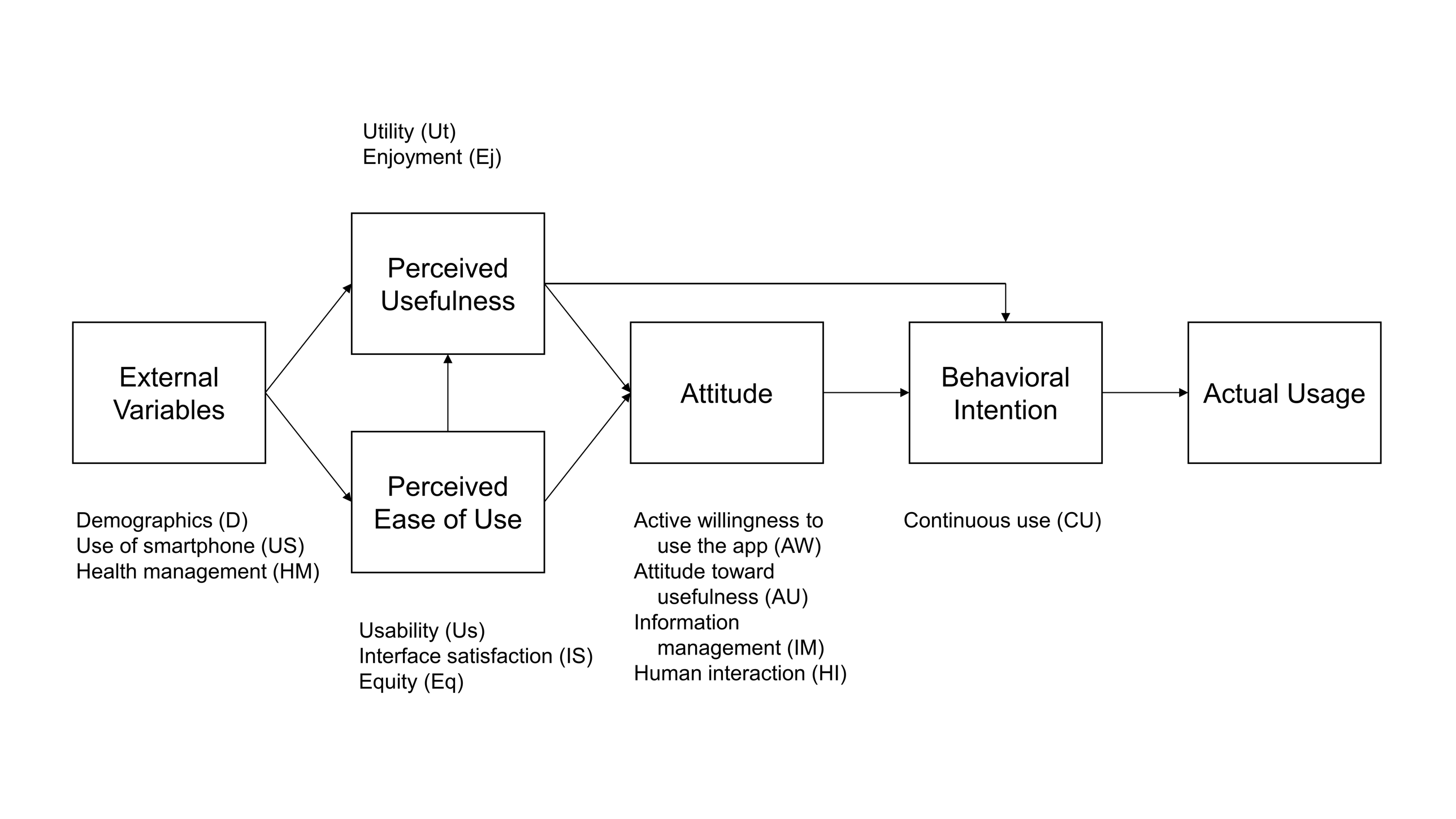
**Supplementary Fig 1.** The matching of each category with Technology Acceptance Model(TAM) variables.

**Supplementary Table 2.** Participant survey response.

| **Variables** | **Total (n=264)** | **Short (n=77)** | **Medium (n=101)** | **Long (n=86)** | ***P*-value** |
| --- | --- | --- | --- | --- | --- |
| **Self-awareness of health** |  |  |  |  | 0.34 |
| Very good | 9 (3.4%) | 1 (1.3%) | 2 (2.0%) | 6 (7.0%) |  |
| Good | 88 (33.3%) | 30 (39.0%) | 31 (30.7%) | 27 (31.4%) |  |
| Average | 143 (54.2%) | 39 (50.6%) | 56 (55.4%) | 48 (55.8%) |  |
| Poor | 24 (9.1%) | 7 (9.1%) | 12 (11.9%) | 5 (5.8%) |  |
| Very Poor | 0 | 0 | 0 | 0 |  |
| **Self-health care (multiple selections possible) ^a^** |  |  |  |  | 0.84 |
| Level 1 (0~2 items) | 67 (25.4%) | 23 (29.9%) | 25 (24.8%) | 19 (22.1%) |  |
| Level 2 (3~5 items) | 163 (61.7%) | 46 (59.7%) | 65 (64.4%) | 52 (60.5%) |  |
| Level 3 (6~ items) | 34 (12.9%) | 8 (10.4%) | 11 (10.9%) | 15 (17.4%) |  |
| **Does your past or current occupation require you to use a smartphone** |  |  |  |  | **0.02** |
| Very likely | 38 (14.4%) | 8 (10.4%) | 17 (16.8%) | 13 (15.1%) |  |
| Likely | 86 (32.6%) | 22 (28.6%) | 42 (41.6%) | 22 (25.6%) |  |
| Neutral | 70 (26.5%) | 21 (27.3%) | 23 (22.8%) | 26 (30.2%) |  |
| Unlikely | 38 (14.4%) | 13 (16.9%) | 9 (8.9%) | 16 (18.6%) |  |
| Not at all likely | 32 (12.1%) | 13 (16.9%) | 10 (9.9%) | 9 (10.5%) |  |
| **What mobile do you currently use? (multiple selections possible)** **^b, c^** |  |  |  |  | 0.81 |
| Level 1 (1 item) | 141 (53.4%) | 48 (62.3%) | 48 (47.5%) | 46 (53.5%) |  |
| Level 2 (2 items) | 84 (31.8%) | 22 (28.6%) | 34 (33.7%) | 29 (33.7%) |  |
| Level 3 (3 items) | 39 (14.8%) | 7 (9.1%) | 19 (18.8%) | 13 (15.1%) |  |
| **How long do you use your smartphone per day, excluding phone calls?** |  |  |  |  | 0.054 |
| Less than 1 hour | 68 (25.8%) | 30 (39.0%) | 23 (22.8%) | 15 (17.4%) |  |
| 1-3 hours | 119 (45.1%) | 27 (35.1%) | 45 (44.6%) | 47 (54.7%) |  |
| 3-5 hours | 49 (18.6%) | 15 (19.5%) | 18 (17.8%) | 16 (18.6%) |  |
| 5 hours or more | 28 (10.6%) | 5 (6.5%) | 15 (14.9%) | 8 (9.3%) |  |
| **What do you most often use your smartphone for, excluding phone calls?** |  |  |  |  | 0.39 |
| Internet | 134 (50.8%) | 34 (44.2%) | 57 (56.4%) | 43 (50.0%) |  |
| Entertainment | 54 (20.5%) | 19 (24.7%) | 19 (18.8%) | 16 (18.6%) |  |
| Social media | 41 (15.5%) | 16 (20.8%) | 12 (11.9%) | 13 (15.1%) |  |
| Learning or work processing | 11 (4.2%) | 4 (5.2%) | 4 (4.0%) | 3 (3.5%) |  |
| Game | 14 (5.3%) | 2 (2.6%) | 6 (5.9%) | 6 (7.0%) |  |
| Other | 10 (3.8%) | 2 (2.6%) | 3 (3.0%) | 5 (5.8%) |  |
| **How skilled do you think you are at using your smartphone?** |  |  |  |  | **0.04** |
| Very good | 10 (3.8%) | 1 (1.3%) | 7 (6.9%) | 2 (2.3%) |  |
| Good | 56 (21.2%) | 17 (22.1%) | 27 (26.7%) | 12 (14.0%) |  |
| Fair | 153 (58.0%) | 40 (51.9%) | 53 (52.5%) | 60 (69.8%) |  |
| Poor | 41 (15.5%) | 15 (19.5%) | 14 (13.9%) | 12 (14.0%) |  |
| Very poor | 4 (1.5%) | 4 (5.2%) | 0 | 0 |  |
| **What categories do you search the most about health? (multiple selections possible) ^d^** |  |  |  |  | 0.80 |
| Level 1 (0~2 items) | 194 (73.5%) | 60 (77.9%) | 71 (70.3%) | 63 (73.3%) |  |
| Level 2 (3~5 items) | 69 (26.1%) | 17 (22.1%) | 30 (29.7%) | 22 (25.6%) |  |
| Level 3 (6 items) | 1 (0.4%) | 0 | 0 | 1 (1.2%) |  |
| **Do you use any other healthcare apps or mobile devices other than the app for this study?** |  |  |  |  | 0.99 |
| Yes | 109 (41.3%) | 32 (41.6%) | 42 (41.6%) | 35 (40.7%) |  |
| No | 155 (58.7%) | 45 (58.4%) | 59 (58.4%) | 51 (59.3%) |  |
| **When using a healthcare app, what else do you consider important besides the app's original purpose?** |  |  |  |  |  |
| **Privacy and Security** |  |  |  |  | 0.89 |
| Very important | 109 (41.3%) | 30 (39.0%) | 40 (39.6%) | 39 (45.3%) |  |
| Important | 97 (36.7%) | 30 (39.0%) | 41 (40.6%) | 26 (30.2%) |  |
| Neutral | 49 (18.6%) | 13 (16.9%) | 18 (17.8%) | 18 (20.9%) |  |
| Low important | 9 (3.4%) | 4 (5.2%) | 2 (2.0%) | 3 (3.5%) |  |
| Not at all important | 0 | 0 | 0 | 0 |  |
| **Counseling or coach's opinion based on my health information** |  |  |  |  | 0.20 |
| Very important | 57 (21.6%) | 11 (14.3%) | 24 (23.8%) | 22 (25.6%) |  |
| Important | 130 (49.2%) | 39 (50.6%) | 51 (50.5%) | 40 (46.5%) |  |
| Neutral | 61 (23.1%) | 23 (29.9%) | 17 (16.8%) | 21 (24.4%) |  |
| Low important | 14 (5.3%) | 3 (3.9%) | 8 (7.9%) | 3 (3.5%) |  |
| Not at all important | 2 (0.8%) | 1 (1.3%) | 1 (1.0%) | 0 |  |
| **How easy is the app to use** |  |  |  |  | 0.27 |
| Very important | 66 (25.0%) | 15 (19.5%) | 23 (22.8%) | 28 (32.6%) |  |
| Important | 107 (40.5%) | 32 (41.6%) | 44 (43.6%) | 31 (36.0%) |  |
| Neutral | 81 (30.7%) | 27 (35.1%) | 31 (30.7%) | 23 (26.7%) |  |
| Less important | 10 (3.8%) | 3 (3.9%) | 3 (3.0%) | 4 (4.7%) |  |
| Not at all important | 0 | 0 | 0 | 0 |  |
| **How many healthcare apps do you usually use?** |  |  |  |  | 0.56 |
| None | 105 (39.8%) | 33 (42.9%) | 37 (36.6%) | 35 (40.7%) |  |
| 1 | 108 (40.9%) | 31 (40.3%) | 42 (41.6%) | 35 (40.7%) |  |
| 2~3 | 48 (18.2%) | 13 (16.9%) | 19 (18.8%) | 16 (18.6%) |  |
| 4 or more | 3 (1.1%) | 0 | 3 (3.0%) | 0 |  |
| **Did you find it easy to use healthcare apps proficiently?** |  |  |  |  | 0.002 |
| Yes | 143 (54.2%) | 28 (36.4%) | 59 (58.4%) | 56 (65.1%) |  |
| No | 95 (36.0%) | 41 (53.2%) | 29 (28.7%) | 25 (29.1%) |  |
| I have never tried | 26 (9.8%) | 8 (10.4%) | 13 (12.9%) | 5 (5.8%) |  |
| **How much time in total do you spend per day to enter your health information when using the healthcare app? Or how much time are you willing to spend?** |  |  |  |  | 0.36 |
| Less than 5 minutes | 112 (42.4%) | 30 (39.0%) | 48 (47.5%) | 34 (39.5%) |  |
| 5-15 minutes | 123 (46.6%) | 40 (51.9%) | 44 (43.6%) | 39 (45.3%) |  |
| 15-30 minutes | 22 (8.3%) | 6 (7.8%) | 6 (5.9%) | 10 (11.6%) |  |
| 30-45 minutes | 5 (1.9%) | 1 (1.3%) | 2 (2.0%) | 2 (2.3%) |  |
| 45 minutes or longer | 2 (0.8%) | 0 | 1 (1.0%) | 1 (1.2%) |  |

Notes: ^a^Self-health care activity item list: (1) Regular exercise, (2) Regular comprehensive health examination, (3) Sufficient sleep and rest, (4) Dietary control (nutrition, calories), (5) Abstention from drinking or smoking cessation, (6) Intake of nutritional supplements or dietary supplements (restorative medicine), (7) Others. Level 1= 0~2 items select, Level 2= 3~5 items select, Level 3= 6 ~ items select

^b^Device item list: (1) Smartphone, mobile phone, (2) Laptop, (3) Tablet, Table PC, (4) None. Level 1= 1 item select, Level 2= 2 items select, Level 3= 3 items select

^c^Excluding the 1 participant with the missing response

^d^Search classification about health item list: (1) Healthy lifestyle habits, (2) Disease-related information, (3) Medicine or healthy functional food, (4) Treatment, (5) Medical institutions or health care providers, (6) Do not search. Level 1= 0~2 items select, Level 2= 3~5 items select, Level 3= 6 items select

The bold values represent statistically significant (*P* < 0.05) comparisons.

**Supplementary Table 3**. Distribution of clustering groups by survey category according to Technology Acceptance Model (TAM) structure.

| **Category** | **Response classification** | **Short-use (n=77)** | **Medium-use (n=101)** | **Long-use (n=86)** | ***P*-value(SM**^a^**)** | ***P*-value(SL**^b^**)** | ***P*-value(ML**^c^**)** | ***P*-value(SML**^d^**)** | **Total (n=264)** |
| --- | --- | --- | --- | --- | --- | --- | --- | --- | --- |
| Usability | High | 40 (51.9%) | 75 (74.3%) | 68 (79.1%) | **< .01** | 0.07 | 0.33 | **0.01** | 183 (69.3%) |
|  | Low | 37 (48.1%) | 26 (25.7%) | 18 (20.9%) |  |  |  |  | 81 (30.7%) |
| Interface satisfaction | High | 45 (58.4%) | 78 (77.2%) | 73 (84.9%) | **0.01** | **< .01** | 0.19 | **< .01** | 197 (74.6%) |
|  | Low | 32 (41.6%) | 23 (22.8%) | 13 (15.1%) |  |  |  |  | 67 (25.4%) |
| Equity | High | 44 (57.1%) | 77 (76.2%) | 72 (83.7%) | **0.01** | **< .01** | 0.20 | **< .01** | 193 (73.1%) |
|  | Low | 33 (42.9%) | 24 (23.8%) | 14 (16.3%) |  |  |  |  | 71 (26.9%) |
| Utility | High | 48 (62.3%) | 78 (77.2%) | 70 (81.4%) | **0.03** | **0.01** | 0.48 | **0.01** | 196 (74.2%) |
|  | Low | 29 (37.7%) | 23 (22.8%) | 16 (18.6%) |  |  |  |  | 68 (25.8%) |
| Enjoyment | High | 23 (29.9%) | 45 (44.6%) | 52 (60.5%) | 0.28 | 0.41 | 0.82 | 0.54 | 120 (45.5%) |
|  | Low | 54 (70.1%) | 56 (55.4%) | 34 (39.5%) |  |  |  |  | 144 (54.5%) |
| Active willingness to use the app | High | 17 (22.1%) | 46 (45.5%) | 52 (60.5%) | 0.97 | 0.75 | 0.70 | 0.92 | 115 (43.6%) |
|  | Low | 60 (77.9%) | 55 (54.5%) | 34 (39.5%) |  |  |  |  | 149 (56.4%) |
| Attitude toward usefulness | High | 45 (58.4%) | 75 (74.3%) | 69 (80.2%) | **0.05** | 0.87 | 0.07 | 0.10 | 189 (71.6%) |
|  | Low | 32 (41.6%) | 26 (25.7%) | 17 (19.8%) |  |  |  |  | 75 (28.4%) |
| Information management | High | 41 (53.2%) | 65 (64.4%) | 62 (72.1%) | 0.18 | 0.92 | 0.21 | 0.32 | 168 (63.6%) |
|  | Low | 36 (46.8%) | 36 (35.6%) | 24 (27.9%) |  |  |  |  | 96 (36.4%) |
| Human interaction | High | 34 (44.2%) | 47 (46.5%) | 54 (62.8%) | 0.75 | **0.02** | **0.03** | **0.03** | 135 (51.1%) |
|  | Low | 43 (55.8%) | 54 (53.5%) | 32 (37.2%) |  |  |  |  | 129 (48.9%) |

Notes: TAM: technology acceptance model.

^a^SM: Short-use, Medium-use. ^b^SL: Short-use, Long-use. ^c^ML: Medium-use, Long-use. ^d^SML: Short-use, Medium-use, Long-use.

The bold values represent statistically significant (***P*** < 0.05) comparisons.

**Supplementary Table 4.** Results from structural equation modelling (SEM) showing the relevance among Technology Acceptance Model (TAM) variables with their estimates, standard error, z-value, and ***P***-value.

| **Path** | | | **Estimate** | **Std.Err** | **z-value** | ***P*-value** |
| --- | --- | --- | --- | --- | --- | --- |
| External Variables | → | (Perceived) Usefulness | -0·061 | 0·056 | -1·099 | 0·272 |
| External Variables | → | Perceived ease-of-use | 0·464 | 0·162 | 2·862 | 0·004 |
| Perceived ease-of-use | → | (Perceived) Usefulness | 0·387 | 0·067 | 5·776 | 0·000 |
| Perceived ease-of-use | → | Attitude | -0·191 | 0·118 | -1·609 | 0·108 |
| (Perceived) Usefulness | → | Attitude | 0·677 | 0·284 | 2·386 | 0·017 |
| (Perceived) Usefulness | → | Behavioural intention | 0·612 | 0·137 | 4·476 | 0·000 |
| Attitude | → | Behavioural intention | 0·184 | 0·051 | 3·594 | 0·000 |
| Motivation | → | (Perceived) Usefulness | 0·533 | 0·182 | 2·926 | 0·003 |
| Motivation | → | Perceived ease-of-use | 0·293 | 0·349 | 0·839 | 0·401 |
| Motivation | → | Behavioural intention | -0·197 | 0·154 | -1·279 | 0·201 |
| Behavioural intention | → | Actual usage | 0·618 | 0·102 | 6·048 | 0·000 |

Notes: Std.Err = standard error.
